# Hypokalemic Periodic Paralysis Syndrome

**DOI:** 10.7759/cureus.109596

**Published:** 2026-05-25

**Authors:** Dhruva Govil, Sonora Desai, Komal Singh, Ali Soueidan

**Affiliations:** 1 Internal Medicine, Henry Ford Providence Southfield Hospital, Southfield, USA; 2 Critical Care Medicine, Henry Ford Providence Southfield Hospital, Southfield, USA

**Keywords:** critical care, hypokalemic periodic paralysis, ion channel dysfunction, neuromuscular diseases, potassium

## Abstract

Hypokalemic periodic paralysis (HPP) is a rare neuromuscular disorder characterized by recurrent episodes of acute muscle weakness or paralysis secondary to hypokalemia. Attacks are often triggered by metabolic or physiologic stressors and may require critical care management. We present two cases of HPP, highlighting challenges in management. A 22-year-old male and a 42-year-old male presented with severe hypokalemia and acute flaccid paralysis requiring intensive care unit admission due to profoundly low potassium levels. Both patients required aggressive repletion with symptom resolution. Despite continued outpatient management, both patients re-presented within one to two months with recurrent episodes requiring repeat hospitalization. HPP results from skeletal muscle ion channel dysfunction leading to episodic paralysis. Management is challenging due to the narrow therapeutic correction window and unpredictable recurrent attacks. These cases highlight the need for standardized protocols and long-term strategies to improve outcomes, along with reducing recurrence and complications.

## Introduction

Hypokalemic periodic paralysis (HypoPP) is a rare inherited skeletal muscle channelopathy that presents with episodic muscle weakness in the setting of low serum potassium. It is most commonly associated with mutations in the CACNA1S and SCN4A genes, which affect calcium and sodium channel function in skeletal muscle fibers [1-3]. With an estimated prevalence of approximately one in 100,000, it is likely underrecognized given its variable presentation and overlap with other causes of acute flaccid paralysis [3].

Clinically, patients often present with sudden, often symmetric weakness that can range from mild functional limitation to complete paralysis. Episodes are frequently triggered by recognizable factors, such as high carbohydrate intake, rest after exertion, or physiologic stress, though triggers are not always identified [4,5]. In severe cases, patients may require intensive care monitoring due to profound hypokalemia and associated critical risks, including life-threatening cardiac arrhythmias and acute respiratory compromise from diaphragmatic weakness. This report highlights the novelty of managing severe, recurrent episodes requiring immediate critical care interventions despite patient compliance with outpatient management.

Despite a well-described genetic basis, management remains inconsistent in practice. While potassium repletion is effective for acute episodes, preventing recurrence is difficult. Response to commonly used therapies, such as acetazolamide, is variable and depends on the underlying genotype, which is not routinely used to guide treatment decisions [4-6]. In addition, recurrent hospitalizations suggest that outpatient management strategies are often insufficient. This case series describes two patients with HypoPP who experienced recurrent severe episodes requiring intensive care admission, highlighting the challenges of maintaining potassium stability outside the hospital and underscoring gaps in long-term disease management.

## Case presentation

Case 1

A 21-year-old male with a past medical history of known familial hypokalemic periodic paralysis presented due to generalized muscle weakness. The patient's initial laboratory evaluation demonstrated profound hypokalemia, prompting an immediate admission to the intensive care unit (ICU) for close cardiac monitoring and aggressive electrolyte management.

Upon presentation, the patient was vitally stable with labs significant for hypokalemia and magnesium of 1.6 mEq/L (Table 1). A standardized neurological examination revealed a complete loss of both proximal and distal motor strength (0/5) in all four extremities, while deep tendon reflexes were diffusely diminished. Sensation remained fully intact throughout. The patient was admitted to the ICU, where he received intensive potassium and magnesium supplementation, consisting of a total of 160 mEq of potassium and 8 g of magnesium.

**Table 1:** Initial lab results (Case 1)

Lab	Value	Reference Range
Potassium (K)	2.4 mmol/L	3.5-5.1 mmol/L
Magnesium (Mg)	1.6 mEq/L	1.3-2.1 mEq/L

After proper repletion, the patient's generalized weakness completely resolved without any further intervention. Upon discharge, the patient was started on scheduled spironolactone, potassium tablets, and acetazolamide. Despite reporting strict compliance with his home medication regimen, the patient presented to the hospital roughly one month later with similar symptoms and was found to have another exacerbation of his hypokalemic paralysis requiring repeat ICU admission.

Case 2

A 41-year-old male with a past medical history of hypokalemic periodic paralysis, which was diagnosed at age 10, presented due to a sudden onset of generalized weakness. On arrival, the patient was unable to ambulate and demonstrated diffuse muscle weakness. Neurological examination confirmed profound, symmetrical proximal and distal weakness in all extremities. The presentation was notable for mild respiratory involvement requiring supplemental oxygen via a nasal cannula; however, bulbar symptoms were entirely absent, and an initial electrocardiogram (ECG) showed no acute arrhythmogenic changes.

On arrival, the patient was vitally stable with labs significant for severe hypokalemia and hypomagnesemia of 1.6 mg/dL (Table 2). Due to the extreme severity of the hypokalemia and the associated high risk of impending cardiac arrhythmias, the patient was immediately admitted to the ICU. He received a total of 250 mEq of potassium and 8 g of magnesium while continuing his home spironolactone.

**Table 2:** Initial lab results (Case 2)

Lab	Value	Reference Range
Potassium (K)	1.6 mmol/L	3.5-5.1 mmol/L
Magnesium (Mg)	1.6 mEq/L	1.3-2.1 mEq/L

The patient's potassium gradually improved with aggressive supplementation. However, due to the extreme lability of cellular potassium uptake in HypoPP, the patient developed severe rebound hyperkalemia within the same day, with his serum potassium peaking rapidly at 6.2 mmol/L. This acute shift was accompanied by hyperkalemic ECG changes, necessitating immediate medical intervention. The patient was treated successfully with temporizing therapies, including intravenous calcium gluconate for myocardial stabilization along with insulin and dextrose to shift potassium back intracellularly, which safely corrected the levels.

The patient was subsequently discharged on home potassium and instructed to take spironolactone as part of his home medication regimen. Unfortunately, the patient presented to the hospital again two months later with recurrent severe weakness, which again required ICU admission and potassium repletion.

**Figure 1:**
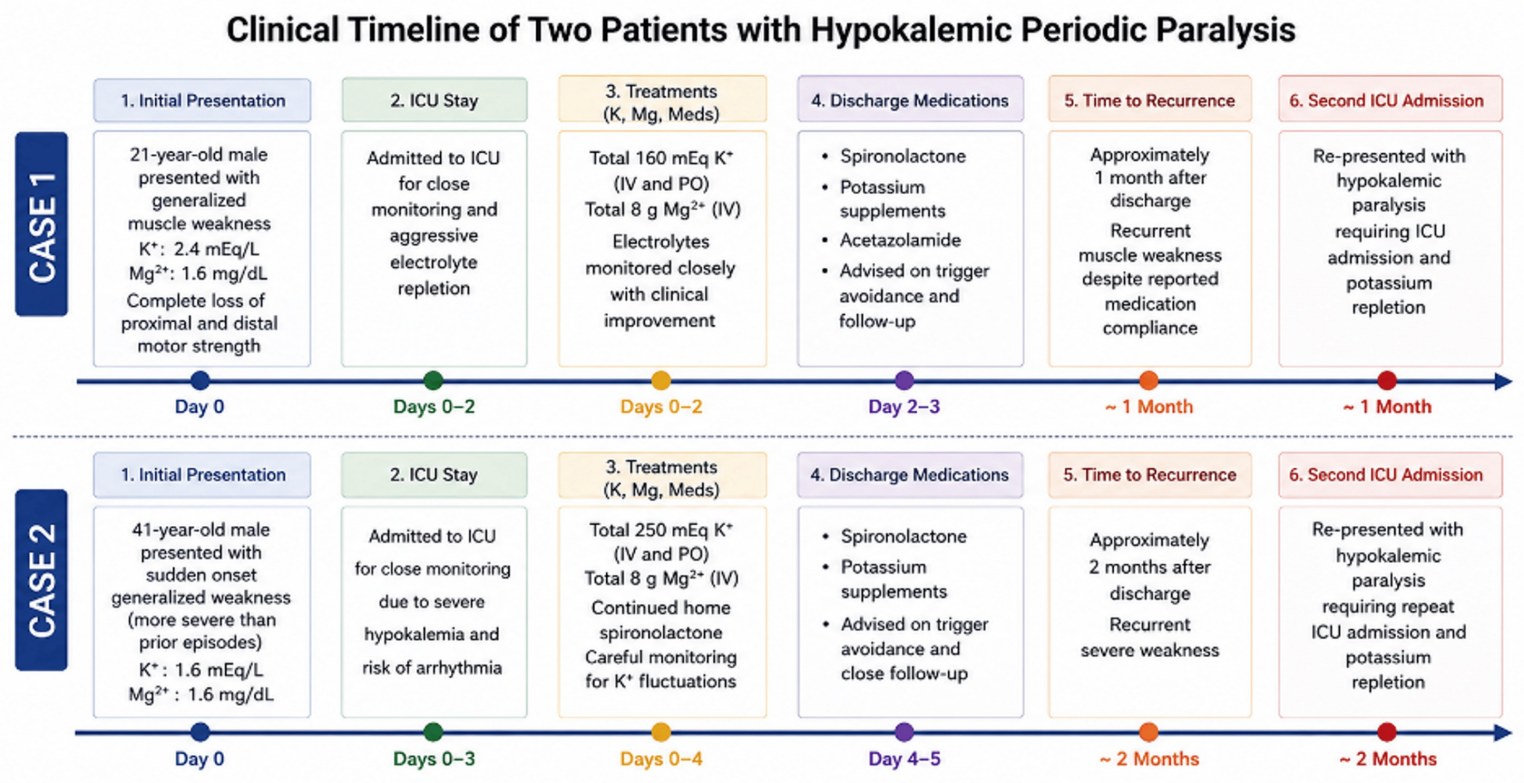
Clinical timeline of both cases Image created by authors using PowerPoint (Microsoft® Corp., Redmond, WA)

The clinical course for both patients is summarized in Figure 1.

## Discussion

HypoPP is a disorder of ion channel dysfunction, with most cases linked to CACNA1S and SCN4A gene mutations. These mutations disrupt the normal conductance of gating pore currents that allow cations (H+ or sodium) to leak through the mutant voltage sensor, leading to aberrant membrane depolarization and episodic symmetrical weakness. While the thyrotoxic HypoPP variant is commonly seen in Asian males, familial HypoPP has no consistent association with race, though male penetrance is significantly higher than female penetrance [1-3]. The clinical presentation of these cases underscores that HypoPP must remain on the differential diagnosis for acute flaccid paralysis regardless of demographic factors, helping clinicians distinguish it from alternative diagnoses, such as Guillain-Barré syndrome, acute spinal cord pathology, or metabolic myopathies.

Management of HypoPP centers on timely potassium replacement and careful monitoring of clinical response. Potassium supplementation is crucial for aborting acute attacks, while carbonic anhydrase inhibitors, such as acetazolamide and dichlorphenamide, are commonly used for prophylaxis. However, clinical response to acetazolamide is variable and genotype-dependent, with approximately 46-50% of patients reporting benefit overall. SCN4A-associated disease demonstrates reduced efficacy and, in some cases, paradoxical worsening, which complicates outpatient management and increases the risk of recurrence [4,5]. This variability highlights the need for genotype-driven treatment approaches in standard practice. Although dichlorphenamide has demonstrated better efficacy in reducing attack frequency in randomized trials, its accessibility is often limited by cost and availability [6].

From a critical care perspective, recurrent ICU admissions often reflect the difficulty of maintaining potassium homeostasis outside the hospital rather than a failure of acute management. Both patients experienced symptom resolution with aggressive electrolyte repletion, yet re-presented within weeks to months, highlighting the narrow therapeutic window of potassium replacement and the challenges of transitioning care safely to the outpatient setting. The clinical course of Case 2 specifically highlights the high risk of rapid, severe rebound hyperkalemia during aggressive repletion. Because the total body potassium deficit is not true depletion but rather a massive intracellular shift, intravenous and oral dosing can easily trigger rapid overcorrection, causing life-threatening hyperkalemic ECG changes that require immediate temporizing therapy. Fluctuating potassium levels further emphasize the need for structured discharge planning, patient education on trigger avoidance, frequent outpatient lab checks, and early subspecialty follow-up to reduce readmissions.

Long-term complications, such as fixed myopathy, reported in up to one-third of patients with longstanding disease, remain poorly understood. For CACNA1S mutations, reduced voltage-dependent calcium release (approximately two-thirds of normal) may contribute to progressive weakness, though this mechanism does not apply to SCN4A-associated disease. Progressive muscle fiber damage with fat replacement can occur even in patients without attacks of paralysis, suggesting that the underlying channelopathy itself, not just recurrent episodes, contributes to the myopathy [7,8].

Emerging therapies aimed at modifying disease mechanisms remain investigational. Experimental approaches in mouse models suggest that reducing NKCC co-transporter activity (with loop diuretics) may reverse acute attacks by promoting hyperpolarization, though this has not been validated in human trials. While sodium-glucose cotransporter 2 (SGLT2) inhibitors have shown some promise in reducing hyperkalemia risk in patients on RAAS inhibitors through enhanced kaliuresis and ENaC activation, there is no published evidence supporting this specifically for HypoPP. Furthermore, antisense oligonucleotides and gene-targeted therapies remain in early investigational stages [9,10]. These cases are primarily hypothesis-generating, demonstrating that HypoPP is a chronic condition marked by critical management limitations outside the hospital setting. Broader use of genetic testing, genotype-informed therapy selection, and standardized long-term management pathways may reduce recurrence and prevent repeated ICU admissions.

## Conclusions

This case series highlights the difficulty of maintaining potassium stability in patients with hypokalemic periodic paralysis despite appropriate inpatient management. Although both patients improved with aggressive electrolyte repletion, each returned within months with recurrent weakness requiring repeat ICU admission, underscoring how challenging it is to manage this condition outside the hospital. In addition, the inconsistent response to standard prophylactic therapies made long-term control even more difficult in these patients. These cases emphasize the need for closer outpatient follow-up, clearer discharge planning, detailed patient counseling on dietary and lifestyle triggers, and a more individualized approach to therapy, particularly in patients with recurrent or severe disease. Without this, repeat hospitalizations and ICU utilization are likely to continue.
